# Cellularity Improvement of EUS-FNB Specimens for Pancreatic Adenocarcinoma Using a Cell-Capturing Scaffold

**DOI:** 10.3390/jcm15218433

**Published:** 2026-10-29

**Authors:** Riccardo Di Pangrazio, Leila Babaiyan, Caterina Talerico, Tiberio Corati, Emanuela Pilozzi, Bruno Annibale, Francesco Panzuto, Marianna Signoretti

**Affiliations:** 1 Digestive Disease Unit, Sant’ Andrea University Hospital, Sapienza University of Rome, 00189 Rome, Italy; riccardo.dipangrazio@uniroma1.it (R.D.P.); leila.babaiyan@uniroma1.it (L.B.); bruno.annibale@uniroma1.it (B.A.); msignorettii@ospedalesantandrea.it (M.S.); 2 Department of Clinical and Molecular Medicine, Sapienza University of Rome, Anatomic Pathology Unit, Sant’Andrea University Hospital, 00189 Rome, Italy; ctalerico@ospedalesantandrea.it (C.T.); tiberio.corati@uniroma1.it (T.C.); emanuela.pilozzi@uniroma1.it (E.P.); 3 Department of Medical-Surgical Science and Translational Medicine, Sapienza University of Rome, Sant’Andrea University Hospital, 00189 Rome, Italy

**Keywords:** pancreatic ductal adenocarcinoma, endoscopic ultrasound, fine-needle biopsy, EUS-FNB, CytoMatrix, cell block, precision oncology

## Abstract

**Background/Objectives:** Molecular profiling is increasingly relevant in pancreatic ductal adenocarcinoma (PDAC), requiring endoscopic ultrasound-guided fine-needle biopsy (EUS-FNB) specimens with adequate tumor cellularity for downstream genomic analyses. Conventional specimen processing methods may be limited by suboptimal cellularity and blood contamination. A cell-capturing scaffold (CCS), a foam-like porous structure designed to retain cytological material, may improve specimen quality and cellular yield. This study aimed to compare histologic adequacy and neoplastic cellularity between CCS-processed and conventional formalin-fixed (FM) EUS-FNB specimens. **Methods:** Consecutive patients who underwent EUS-guided tissue acquisition for solid pancreatic lesions between November 2023 and June 2024 were retrospectively included. Tissue acquisition was performed using a 22-gauge Franseen-tip FNB needle. The first needle pass was processed using CCS, while subsequent passes were dispersed in formalin. Histologic adequacy, microfragment length and neoplastic cellularity were assessed by two expert pancreatic pathologists using dedicated scoring systems. **Results**: Twenty patients were included, with diagnoses of PDAC. Histologic core material was obtained in 17 CCS-processed and 15 FM samples, with no significant difference in histologic adequacy or microfragment length between groups (*p* = 0.414 and *p* = 0.276, respectively). CCS-processed samples showed a trend toward higher neoplastic cellularity compared with FM specimens (*p* = 0.0506). **Conclusions**: In this retrospective pilot study, CCS processing was associated with a trend toward higher neoplastic cellularity in EUS-FNB specimens from patients with PDAC, with comparable histologic adequacy. Further prospective studies are warranted to confirm these results and to evaluate the potential role of CCS in molecular analyses of pancreatic EUS-FNB specimens.

## 1. Introduction

**Figure 1:**
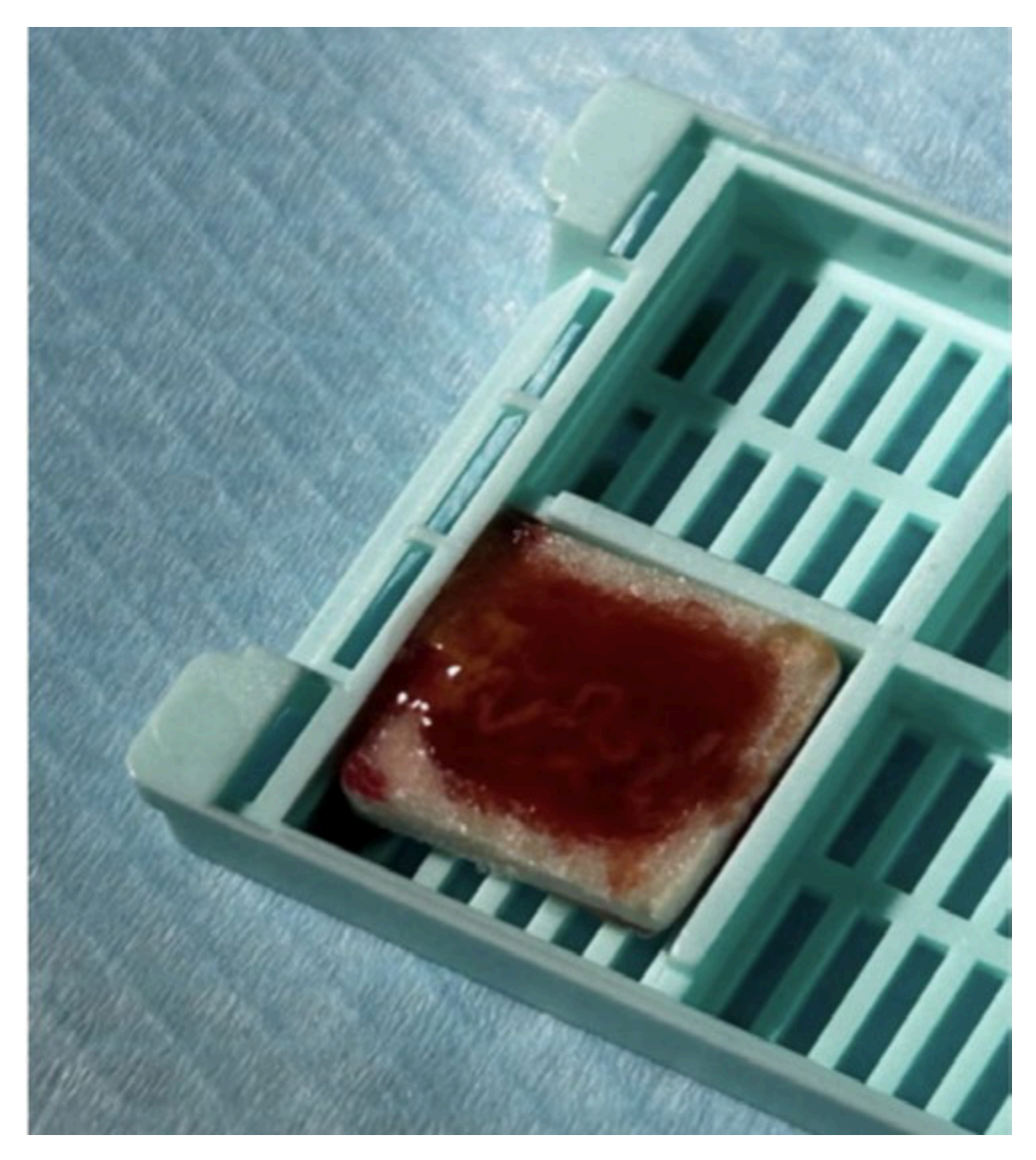
Representative image of the cell-capturing scaffold (CCS) containing endoscopic ultrasound-guided fine-needle biopsy (EUS-FNB) specimen material prior to histologic processing.

Pancreatic cancer is one of the leading causes of cancer-related mortality in Europe, with an estimated incidence of approximately 8 cases per 100,000 individuals [1,2]. The global burden of pancreatic ductal adenocarcinoma (PDAC) is increasing, partly due to the rising prevalence of risk factors such as smoking, alcohol consumption, and chronic pancreatitis, as well as genetic predisposition in a subset of patients [3,4]. Because PDAC is frequently diagnosed at an advanced stage, curative surgical resection is rarely feasible and systemic therapy remains the mainstay of treatment. In this context, molecular profiling has become a key component of precision oncology, enabling the identification of actionable genomic alterations and supporting individualized therapeutic strategies [5]. Although targeted therapies are currently limited to selected molecular subgroups, international guidelines recommend tumor molecular characterization in patients eligible for systemic treatment [6,7]. PDAC is characterized by a complex mutational landscape, with KRAS mutations occurring in the majority of cases and frequent alterations in tumor suppressor genes such as TP53, CDKN2A and SMAD4, as well as in DNA damage repair pathways and chromatin remodeling genes [8]. Endoscopic ultrasound-guided tissue acquisition (EUS-TA) represents the standard approach for sampling solid pancreatic lesions [9]. In recent years, fine-needle biopsy (FNB) needles have progressively replaced fine-needle aspiration (FNA), as they provide higher diagnostic performance, improved preservation of tissue architecture, and reduced dependence on rapid on-site evaluation (ROSE) [9,10,11]. EUS-FNA is limited by its reliance on ROSE by an experienced cytopathologist and its reduced ability to obtain histologic core tissue (≥550 μm), defined as an intact tissue fragment, which is increasingly relevant in the era of precision oncology [9,12]. FNB provides higher diagnostic accuracy and allows acquisition of histologic core tissue with fewer needle passes compared with FNA [9,10,11], while enabling the collection of adequate cellular material and nucleic acids for molecular analyses [13]. Several studies have demonstrated the feasibility of extracting high-quality DNA and RNA from EUS-guided fine-needle biopsy (EUS-FNB) specimens in patients with PDAC [14,15]. In addition, FNB has been reported to provide higher tissue adequacy for microsatellite instability evaluation compared with FNA (88.9% vs. 35.7%, *p* = 0.03) [16]. In a prospective pilot study, Carrara et al. reported successful DNA extraction and subsequent next-generation sequencing in the majority of EUS-FNB samples, with comparable diagnostic yield between cytology and histology specimens (88% vs. 91%) [17]. Furthermore, next-generation sequencing can also be performed on residual liquid-based cytology specimens, with studies demonstrating the feasibility of this approach [18]. EUS-derived specimens can be processed using different techniques, including direct smears, liquid-based cytology and cell block (CB) preparation [19]. With FNB needles, the sample is macroscopically visible and can be directly placed in formalin, similarly to standard biopsies [20]. Among these, CBs allow preservation of tissue architecture and enable immunocytochemical and molecular analyses [21,22,23]. Genomic analyses are typically performed on formalin-fixed paraffin-embedded (FFPE) material and/or liquid-based cytology [17]. Recently, a novel CB approach based on a cell-capturing scaffold (CCS) has been introduced (Figure 1).

This porous scaffold is designed to capture and retain cytologic material through its porosity and net positive surface charge, improving specimen handling, sectioning, staining, and potentially enhancing diagnostic and molecular utility [21,22,23]. Evidence from other tumor types such as lung, urinary and skin cancers suggests that CCS may allow the generation of multiple sections from limited material, improving immunocytochemical analyses, confocal imaging, and DNA and RNA extraction [21,22,23]. CCS-based specimens have also been applied to ex vivo fluorescence confocal microscopy (FCM), allowing rapid digital microscopic evaluation of fresh, unfixed samples without conventional slide preparation [24]. Preliminary data in pancreatic EUS-FNA/FNB specimens have shown promising histologic adequacy rates (up to 93.8%) [25]. The use of a naturally derived, porous scaffold for the histological evaluation of samples after FNB of pancreatic adenocarcinoma, in terms of diagnostic adequacy, histological core and sample cellularity, has never been assessed. Therefore, the primary endpoint of this study was the comparison of neoplastic cellularity between CCS-processed and formalin-fixed (FM) samples. Secondary endpoints included histologic adequacy and microfragment length. 

## 2. Materials and Methods

This is a retrospective, single-center, pilot study, including consecutive patients who underwent EUS-TA for solid pancreatic lesions between November 2023 and June 2024. The CCS was available at our institution during the study period, when it was routinely employed for specimen processing in all consecutive patients undergoing EUS-FNB of solid lesions. Patients with a final diagnosis other than PDAC or with inadequate histological sampling were excluded from the analysis. A single experienced endosonographer performed all procedures under deep sedation with spontaneous breathing. A linear-array echoendoscope (GF-UCT180; Olympus Medical Systems, Tokyo, Japan) coupled with an ultrasound processor (Aloka ProSound Alpha 10; Hitachi Aloka Medical, Tokyo, Japan) was used. Tissue acquisition was performed using a 22-gauge Franseen-tip fine-needle biopsy (FNB) needle (Acquire™; Boston Scientific, Marlborough, MA, USA). For each lesion, two to three passes were performed. The first pass was entirely processed using the CCS (CytoMatrix^®^; CE-IVD, UCS Diagnostics, Morlupo, Italy), consisting of a foam-like porous substrate that retains cytologic material through its porosity and net positive surface charge [25] (Figure 1). The subsequent passes were placed in pre-filled formalin vials as part of standard clinical practice. When macroscopic on-site evaluation (MOSE) indicated inadequate material, a third pass was performed and placed in formalin, pooled with the second pass for histologic processing as a single FM specimen.

### 2.1. Specimen Processing and Evaluation

Both CCS and FM samples were processed, as part of routine specimen handling during the study period, according to the same protocol: 12 h fixation in buffered formalin, automated processing using a Donatello Diapath tissue processor (Diapath S.p.A., Bergamo, Italy), manual paraffin embedding, sectioning and hematoxylin–eosin (H&E) staining. All samples were evaluated jointly by two expert pancreatic pathologists (E.P., C.T.), who assigned a single consensus score to each specimen.

### 2.2. Histologic Assessment

**Figure 2:**
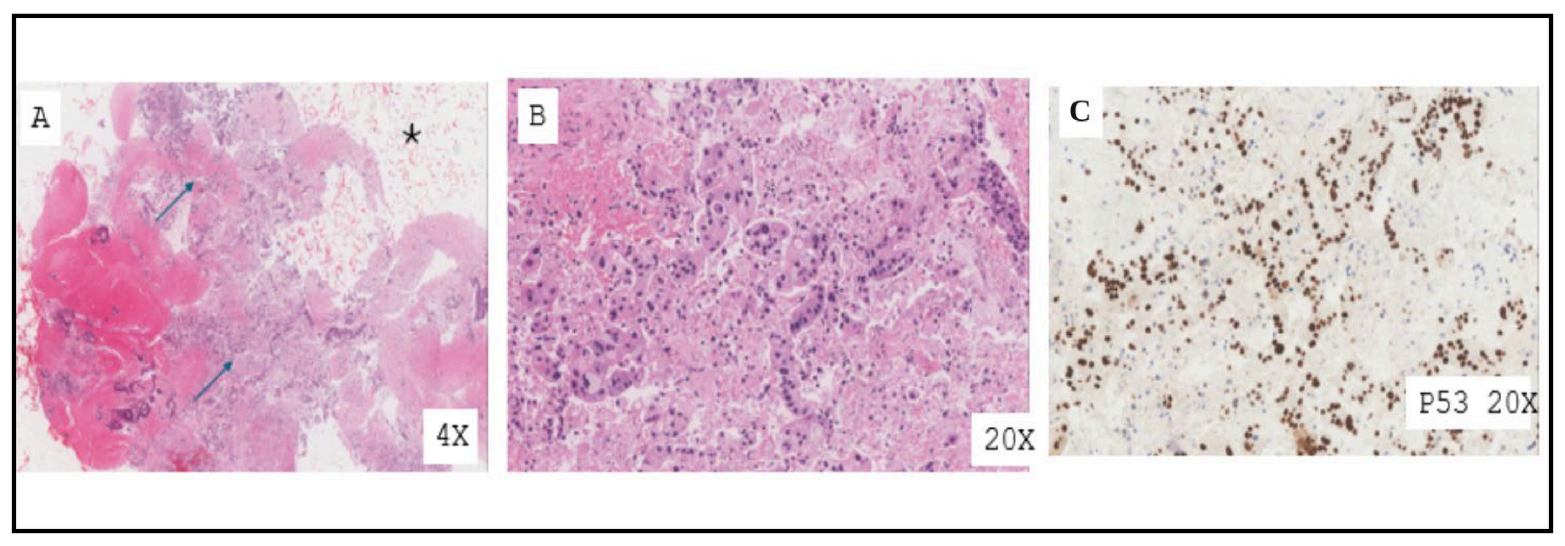
Representative histologic section of a CCS-processed specimen from pancreatic ductal adenocarcinoma showing neoplastic cells. Tumor cells demonstrated p53 positivity on immunohistochemistry. (**A**) Micro-fragments and glands of pancreatic ductal adenocarcinoma (arrows) in cytomatrix net (star) (**B**) Fragments of neoplastic glands (**C**) Strong nuclear expression of P53.

Specimen quality was assessed on H&E-stained sections. Histologic adequacy and neoplastic cellularity were evaluated for each sample using dedicated scoring systems (Table 1 and Table 2). Histologic adequacy was graded according to the number of microfragments measuring ≥0.5 mm; specimens in which no tissue diagnostic for tumor could be identified were scored as insufficient (score 0), regardless of fragment length. Microfragment length was measured on H&E-stained sections and expressed as the sum of the lengths of all tissue fragments in the evaluated specimen. Neoplastic cellularity was assessed by counting neoplastic cells at 400× magnification on one H&E-stained section per specimen; representative cells were defined as neoplastic epithelial cells only, identified based on cytomorphology, with cytokeratin immunostaining performed in doubtful cases. Specimens consisting only of blood clot were scored as acellular (score 1). Representative examples are shown in Figure 2.

Immunohistochemistry was performed when required to support the histopathological diagnosis (e.g., endocrine differentiation, atypical ductal features, or suspected metastasis). Immunostaining was carried out using an automated immunostainer (Bond Max; Leica Biosystems, Wetzlar, Germany) according to the manufacturer’s instructions. Differences between paired CCS and FM samples were analyzed using the Wilcoxon signed-rank test, and the effect size was expressed as the median difference between paired samples (Hodges–Lehmann estimate) with a 95% confidence interval (CI). A sensitivity analysis restricted to patients who underwent two needle passes was also performed. Statistical analyses were performed using MedCalc software version 23 (MedCalc Software Ltd., Ostend, Belgium). A two-sided *p*-value < 0.05 was considered statistically significant. During the preparation of this work, the authors used Claude AI (Claude Sonnet 5) for linguistic and editorial revision and to assist in the review and presentation of the au-thors’ own validated data. The AI tool was not used to generate or modify the original scientific data. Following the use of this tool, the authors reviewed and edited the con-tent as appropriate and take full responsibility for the content of the published article. The study was conducted in accordance with the Declaration of Helsinki, and the pro-tocol was part of the study approved by the Ethics Committee Lazio Area 1 (protocol code CE 0537/2026) on 22 June 2026.

## 3. Results

Twenty-eight consecutive patients underwent EUS-FNB of solid pancreatic lesions during the study period; 8 were excluded because of inadequate histological sampling (n = 4) or a final diagnosis other than PDAC (neuroendocrine neoplasm, n = 2; gastrointestinal stromal tumor, n = 1; inflammatory lesion, n = 1). A total of 20 patients were included, with a median age of 72 years (range 42–84); 14 were male (70%), 6 were female (30%). Lesions were predominantly located in the pancreatic head (17 lesions, 85%); 2 lesions were located in the body (10%) and 1 in the tail (5%) (Table 3).

A final diagnosis of PDAC was established at the index procedure in 19/20 cases, while the remaining case required repeat sampling to obtain diagnostic material. In the patient whose index procedure was non-diagnostic, only the CCS and FM specimens obtained at the diagnostic repeat procedure were included in the comparative analysis. A third needle pass was performed in 8/20 (40%) patients, while the remaining 12 (60%) underwent two passes. The distribution of neoplastic cellularity and histologic adequacy scores is reported in Table 4. The median cellularity score was 2.5 (range 1–3) in CCS-processed samples and 2 (range 1–3) in FM samples. The cellularity score was higher in the CCS sample in 10 patients, equal in 8 and lower in 2. Moderate cellularity (100–500 neoplastic cells) was observed in 10/20 CCS-processed and 3/20 FM samples; no sample showed high cellularity. The median difference in cellularity score between paired CCS and FM samples was 0.5 points (95% CI 0–1; *p* = 0.0506). Histologic core material was obtained in 17 CCS-processed and 15 FM samples; the median histologic score was 2 (range 0–2) in both groups (*p* = 0.414). The median microfragment length was 3 mm (range 0–7 mm) in CCS-processed samples and 4 mm (range 0–10 mm) in FM samples (*p* = 0.276). In the sensitivity analysis restricted to the 12 patients who underwent two passes, the median cellularity score was 2 (range 1–3) in both groups; the score was higher with CCS in 5 patients, equal in 6 and lower in 1 (median difference 0.5 points, 95% CI 0–1; *p* = 0.219). The median histologic score was 2 (range 0–2) in both groups (*p* = 1.0), and the median microfragment length was 4 mm in both groups (CCS: range 0–7 mm; FM: range 0–10 mm; *p* = 0.610). Among the 8 patients who underwent three passes, the median cellularity score was 3 (range 1–3) with CCS and 1.5 (range 1–3) with FM; the score was higher with CCS in 5 patients, equal in 2 and lower in 1 (median difference 1 point, 95% CI −0.5 to 2; *p* = 0.25).

## 4. Discussion

In this study, we evaluated the qualitative and quantitative characteristics of tissue obtained using a 22-gauge Franseen-tip EUS-FNB needle, comparing specimens processed with the CCS and those dispersed in FM. While overall histologic adequacy and microfragment length were comparable between the two processing approaches, CCS was associated with a trend toward higher neoplastic cellularity; however, the difference did not reach statistical significance. Although this observation may suggest a potential advantage for downstream molecular applications, this hypothesis was not tested in the present study and requires validation. In our study, the comparable microfragment length obtained with CCS and formalin-dispersed samples suggests that CCS processing does not compromise tissue architecture; however, the absence of a statistically significant difference does not establish equivalence, and this study was not designed or powered to demonstrate it. The main difference between the two methods was observed in neoplastic cell yield. While most formalin-dispersed samples showed scant cellularity (<100 cells) or were acellular (17/20), moderate cellularity was observed in half of CCS-processed samples (10/20 vs. 3/20), although no sample in either group reached high cellularity. In this context, cellularity represents not only a specimen quality indicator but also a key enabling factor for integrated morphologic and molecular evaluation in pancreatic cancer. This observation is in line with previous reports suggesting that the porous, positively charged structure of the CCS enhances capture, concentration and retention of cytological material, resulting in improved specimen handling and sectioning quality [21,22,23]. Similar results have been described by Montella et al. [23] in ultrasound-guided fine-needle aspiration cytology of metastatic melanoma, where CCS-based samples demonstrated preserved morphology and high cellularity compared with conventional cell block techniques, while also allowing successful downstream molecular analysis. These features may be particularly relevant in PDAC, where the quantity of neoplastic cells is a critical determinant for successful genomic analyses, including next-generation sequencing. However, since DNA/RNA yield and sequencing performance were not assessed in the present study, any benefit of CCS for the molecular profiling of EUS-FNB specimens remains a hypothesis that needs to be tested in dedicated studies. Moreover, even with CCS processing, no sample exceeded 500 neoplastic cells, and whether the observed gain in cellularity is sufficient to improve the success rate of next-generation sequencing remains to be established. This study has some limitations. The small sample size and the single-center retrospective design limit the statistical power and generalizability of the findings, which should therefore be regarded as preliminary; however, the paired within-patient comparison allowed a direct assessment of cellularity differences between CCS- and FM-processed samples. In addition, the analysis was restricted to patients with a final diagnosis of PDAC and patients with inadequate sampling were excluded, which may limit the applicability of our findings to the overall population undergoing EUS-FNB of solid pancreatic lesions. Additionally, the order of needle-pass allocation was fixed and not randomized, as the first pass was consistently assigned to CCS processing and subsequent passes to formalin fixation, reflecting the routine workflow adopted during the study period. Consequently, the observed difference in neoplastic cellularity cannot be attributed to the processing method alone and may partly reflect differences in tissue yield or tumor cellularity between sequential passes. However, data on whether the first FNB pass yields higher neoplastic cellularity than subsequent passes are currently lacking. Furthermore, in the 8 patients who underwent three passes, the FM specimen included material from two passes, which would, if anything, have favored the FM arm; nevertheless, CCS-processed samples still showed higher cellularity in most cases (5/8; median score 3 vs. 1.5), suggesting that the difference observed is unlikely to be explained by the amount of material collected. A sensitivity analysis restricted to patients who underwent two passes also yielded an effect of similar magnitude, although the small size of this subgroup precluded statistical significance. A more robust comparative design would consist of dividing comparable material obtained from the same needle pass between the two processing methods, thereby minimizing the variability related to the sequence of passes; a prospective study adopting this design is warranted to confirm our findings. Since specimens were assessed jointly by the two pathologists and a single consensus score was assigned, interobserver agreement could not be quantified. Moreover, the pathologists were not blinded to the processing method. Finally, neoplastic cellularity was assessed with a semi-quantitative scale on a single H&E-stained section, and the percentage of tumor cells relative to total nucleated cells, a parameter commonly used to define adequacy for molecular testing, was not evaluated.

## 5. Conclusions

In this retrospective pilot study, EUS-FNB provided histologic tissue of good adequacy in patients with PDAC, consistent with previous evidence. Specimens processed using the CCS showed a trend toward higher neoplastic cellularity compared with passes dispersed in FM, while histologic adequacy and microfragment length were comparable between the two approaches. Given the small sample size, the fixed order of pass allocation and the borderline statistical result, these findings should be considered preliminary. Since the quantity of neoplastic cells is a key determinant for the success of genomic assays such as next-generation sequencing, the hypothesis that CCS processing may improve the suitability of EUS-FNB samples for molecular analyses deserves evaluation in adequately powered prospective studies, directly assessing nucleic acid yield and sequencing success.

## Figures and Tables

**Table 1:** Histologic adequacy scoring system used for evaluation of EUS-FNB specimens.

Score	Definition
0	Insufficient material (no tissue diagnostic for tumor)
1	Microfragments < 0.5 mm
2	1–5 microfragments ≥ 0.5 mm
3	6–10 microfragments ≥ 0.5 mm

**Table 2:** Neoplastic cellularity scoring system used for histologic assessment of EUS-FNB specimens. Representative cells refer to neoplastic epithelial cells only.

Score	Definition
1	Acellular sample (absence or minimal representative cells)
2	Scant cellularity (<100 representative cells)
3	Moderate cellularity (100–500 representative cells)
4	High cellularity (>500 representative cells)

**Table 3:** Baseline demographic and lesion characteristics of the study population.

Characteristic	
Number of patients	20
Age, median (range), years	72 (42–84)
Sex, n (%)	
Male	14 (70%)
Female	6 (30%)
Lesion location, n (%)	
Head	17 (85%)
Body	2 (10%)
Tail	1 (5%)
Lesion size, mm	
Mean (range)	28 (17–75)
≤30 mm	12 (60%)
>30 mm	8 (40%)

**Table 4:** Distribution of neoplastic cellularity and histologic adequacy scores in paired CCS-processed and formalin-fixed (FM) specimens. Data are n (%).

Score	CCS (n = 20)	FM (n = 20)
Neoplastic cellularity		
1—Acellular	3 (15%)	6 (30%)
2—Scant cellularity (<100 neoplastic cells)	7 (35%)	11 (55%)
3—Moderate cellularity (100–500 neoplastic cells)	10 (50%)	3 (15%)
4—High cellularity (>500 neoplastic cells)	0	0
Median (range)	2.5 (1–3)	2 (1–3)
Histologic adequacy		
0—Insufficient material	3 (15%)	5 (25%)
1—Microfragments < 0.5 mm	0	0
2—1–5 microfragments ≥ 0.5 mm	17 (85%)	15 (75%)
3—6–10 microfragments ≥ 0.5 mm	0	0
Median (range)	2 (0–2)	2 (0–2)

## Data Availability

The data presented in this study are available on request from the corresponding author due to institutional and privacy restrictions concerning patient-derived clinical and histopathological data.
